# Determination of Serum Levels of Interleukin-6 and the Trace Element Zinc According to the Clinical Status of Patients With COVID-19

**DOI:** 10.1155/ipid/6486467

**Published:** 2025-06-12

**Authors:** Andrea Roman-Pimentel, Sandra Medina-Cáceres, Juana del Valle-Mendoza, Miguel Angel Aguilar-Luis, Sungmin Kym, Ronald Aquino-Ortega, Yordi Tarazona-Castro, Hugo Carrillo-Ng, Eliezer Bonifacio-Velez de Villa, Wilmer Silva-Caso

**Affiliations:** ^1^School of Medicine, Faculty of Health Sciences, Peruvian University of Applied Sciences, Lima, Peru; ^2^Biomedicine Laboratory, Research Center of the Faculty of Health Sciences, Peruvian University of Applied Sciences, Lima, Peru; ^3^Division of Infectious Diseases, Department of Internal Medicine, Chungnam National University School of Medicine, Daejeon, Republic of Korea; ^4^School of Biology, Faculty of Health Sciences, Peruvian University of Applied Sciences, Lima, Peru

**Keywords:** cytokines, IL-6, pandemic, SARS-CoV-2, zinc

## Abstract

In the context of SARS-CoV-2 infection, the present study aimed to determine the clinical and laboratory characteristics and serum levels of IL-6 and zinc in patients with COVID-19 according to their clinical condition in a hospital in Lima, Peru. Patients were divided into 4 groups according to the clinical condition of the disease, the group of patients hospitalized in the intensive care unit, hospitalized patients who did not require intensive care unit, COVID-19 patients who did not require hospitalization, and a control group. It was determined that 64.8% of the patients evaluated were men. Patients hospitalized in the ICU were 11.25 times more likely to have a cough and 36.7 times more likely to have a fever compared to the control group. In the group of hospitalized patients who did not require ICU, the presence of cough was 9.44 times higher than in the control group. The lowest IL-6 values were obtained in the group of COVID-19 patients who did not require hospitalization (2 pg/mL) and the highest in the ICU group (168.5 pg/mL). On the other hand, the highest values of the micronutrient zinc were also obtained in the ICU group (3402.5 μg/dL). In this group, the highest values of lymphocytes, C-reactive protein, and lactate dehydrogenase were also found with statistical significance compared to the group of hospitalized patients who did not require ICU. In conclusion, patients with COVID-19 in the ICU had higher levels of IL-6 and zinc compared to the other groups. This group also had the highest levels of lymphocytes, C-reactive protein, and lactate dehydrogenase compared to the group of hospitalized patients who did not require ICU care.

## 1. Introduction

Clinical presentations of patients with SARS-CoV-2 have been described to range from mild or asymptomatic upper respiratory tract infections that do not require hospitalization to acute respiratory failure with bilateral pulmonary infiltrates requiring mechanical ventilation in intensive care units. In this context, inflammatory markers can be measured in patients to characterize disease severity [1–3]. Some cases have a more severe presentation, which is characterized by a dysregulated immune response due to increased proinflammatory cytokines, such as interleukin-6 (IL-6), IL-1, TNF-α, and IL-12 [4]. The disease can lead to acute respiratory failure, multiple organ dysfunction, and death [5].

Among the known cytokines, IL-6 is considered one of the most important predictive markers of severity and determines the need for mechanical ventilation in patients with COVID-19 [6, 7]. The unregulated elevation of this cytokine alters the normal process of cell cytolysis and apoptosis, which makes it difficult to adequately eliminate the virus [8]. In this way, it contributes to pathological effects such as acute and chronic inflammation [9]. For this reason, drugs that target this molecule, such as tocilizumab, have been studied as part of the treatment protocols for patients with severe COVID-19, although with discordant results that depend on the type of population studied [10, 11].

Research is currently suggesting the use of other anti-inflammatory agents for the treatment of COVID-19. One of these agents is the trace element zinc, which has been studied as an inflammatory mediator of cytokines, particularly IL-6 [12, 13]. Zinc homeostasis in the body is involved in multiple important aspects of the immune system, including cell maturation, differentiation, and proper immune response [14]. Moreover, zinc deficiency can cause imbalanced immune reactions, which can predispose to serious infections [15].

Furthermore, zinc could play an important role in preventing SARS-CoV-2 virus entry by maintaining the integrity of the epithelial barrier and mucous membrane, as well as inhibiting ACE2 activity [16]. Therefore, the objective of this study was to determine the clinical characteristics and serum levels of IL-6 and the micronutrient zinc as biomarkers in patients with COVID-19 according to their hospital care conditions in Lima, Peru.

## 2. Materials and Methods

### 2.1. Study Design and Subjects

A cross-sectional observational study was carried out in two health facilities during the year 2021. Hospitalized patients were recruited from a specialized hospital and patients who did not require hospitalization were recruited from the Biomedicine laboratory of the Peruvian University of Applied Sciences in Lima, Peru. Patients were divided into 4 groups according to the level of hospital care and severity of illness. That is, the group of hospitalized patients in the intensive care unit, hospitalized patients who did not require an intensive care unit, the group of COVID-19 patients who did not require hospitalization, and a control group of healthy individuals.

### 2.2. Inclusion Criteria

The inclusion criteria included the following: confirmed diagnosis of COVID-19, age ≥ 18 years, adults with the capacity to give informed consent, complete clinical and laboratory records of interest, and signed informed consent form.

### 2.3. Exclusion Criteria

The exclusion criteria included the following: previous treatment with cytokine modulators or zinc supplements before sample collection, history of decompensated chronic diseases, immunosuppression, pregnancy or breastfeeding, severe coagulopathies, or contraindications for blood collection.

Participants in the unmatched control group were volunteers with a negative COVID-19 test who came to the Biomedicine laboratory to obtain a SARS-CoV-2 test due to work or travel requirements. They voluntarily agreed to participate in the study and signed their informed consent.

### 2.4. Data Collection

Clinical data such as cough and fever and laboratory data such as complete blood count and C-reactive protein (CRP) were collected on a data collection form by reviewing hospital admission forms, medical records, and laboratory reports by the attending physician. Variables of interest were recorded in a Microsoft Excel spreadsheet using double entry. Blood samples from patients treated at the hospital were obtained within 48 h of admission to reflect the initial stage of the disease. Blood samples from the control group were obtained on the same day as the nasopharyngeal swab after signing the informed consent form.

### 2.5. Identification of SARS-CoV-2

To identify the presence of SARS-CoV-2, a nasopharyngeal swab was obtained from patients with clinical suspicion of COVID-19 who presented symptoms such as cough, fever, fatigue, and respiratory distress, among others, as well as healthy controls that were conducted to rule out infection. Real-time RT-PCR was performed as a standardized diagnostic technique in accordance with the Peruvian national guidelines [17].

### 2.6. Determination of Serum Levels of IL-6 and Zinc

Serum samples were obtained from participants to determine IL-6 and zinc levels. Regarding IL-6 levels, the Human IL-6 ELISA kit (ab178013) (Abcam, Cambridge, UK) was used according to the manufacturer's instructions [18]. As per zinc levels, we used the colorimetric zinc assay kit (ab102507) (Abcam, Cambridge, UK) according to the manufacturer's instructions [19].

Hematological and biochemical analyses were performed according to the protocols established in the hospital where the patients requiring hospitalization were recruited [20].

### 2.7. Statistical Analysis

For characterizing the population, as well as the concentrations of zinc (μg/dL) and IL-6 (pg/mL), a descriptive statistical analysis was carried out with the data provided, using the measures of central tendency and frequency distribution. The normality of the variables was evaluated using the Shapiro–Wilk tests, and the homogeneity of variance of the quantitative variables using the Levene's test.

The ANOVA test was used to compare constant data with normally distributed data, and the Kruskal–Wallis test was used to compare constant data with non-normally distributed data, followed by Dunn's post hoc test for multiple comparisons. We performed these tests to determine if there was a difference between the ICU cases, non-ICU cases, outpatient cases, and control group with respect to IL-6 and zinc.

In addition, multinomial logistic regressions were used to analyze risk factors for which univariate and multivariate analyses were conducted. A variable with a *p* value less than 0.2 in the univariate analysis was included in the multivariate analysis. *p* values less than 0.05 were considered statistically significant.

### 2.8. Ethics Statement

The study protocol was approved by the Ethics Board of the Universidad Peruana de Ciencias Aplicadas (no. FCS-CEI/340-05-21). Samples were collected after the written informed consent was signed to agree to participate. Informed consent was obtained from patients in each group analyzed upon admission to the hospital or laboratory, as appropriate, and was obtained by the attending physician, following the recommendations of the international ethical guidelines for health-related research in human subjects developed by the Council for International Organizations of Medical Sciences (CIOMS), under the auspices of the WHO and UNESCO.

## 3. Results

The participants were divided into four groups, two of which were hospitalized and two not. Among those hospitalized, 26 people required ICU and 26 did not require ICU. Among those not hospitalized, 24 individuals belonged to the group of COVID-19 patients who did not require hospitalization and 12 to the control group.  Table 1 shows the demographic characteristics of the study subjects. Patients were divided according to the following age ranges: young adults (18–29 years), adults (30–59), and older adults (over 60 years). Most patients were in the age group of 30–59 years, followed by those older than 60 years. Similarly, most of the patients were male. According to the univariate analysis, male patients are more likely to be hospitalized: ICU (OR = 6.6, *p* value = 0.014) and non-ICU (OR = 8.4, *p* value = 0.007).

Regarding the clinical presentation and the presence of comorbidities for each evaluated group, the most frequent symptoms described in the group of ICU patients were fever (76.9%), cough (69.2%), and fatigue (46.1%). In the case of hospitalized patients who were not in the ICU, the predominant symptoms were dyspnea (80.7%), cough (65.3%), and fatigue (50.0%). On the other hand, patients in the nonhospitalized groups presented fewer symptoms, the most frequent of which were headache, fever, and cough. Finally, comorbidities were described more frequently in hospitalized patients, including obesity, diabetes mellitus, and arterial hypertension (Table 2). The univariate and multivariate analyses revealed that patients hospitalized in the ICU had 11.25 times higher odds of cough (OR = 11.25, *p* value = 0.006) and 36.7 times higher odds of fever (OR = 36.7, *p* value = 0.02) than the control group. In the non-ICU hospitalized group, cough incidence was 9.44 times higher (OR = 9.44, *p* value = 0.010) than in the control group.

**Figure 1:**
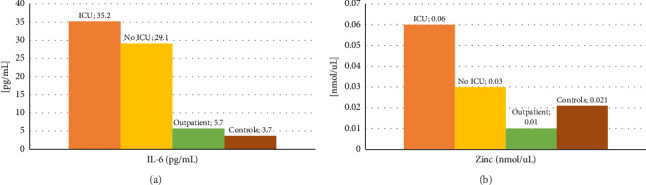
Mean IL-6 and zinc concentrations among different patient groups.

Figure 1 shows the mean concentration values of IL-6 and zinc among the different groups of patients, where higher levels of IL-6 and zinc stand out in the group of those hospitalized in the ICU, and lower values of IL-6 in the control group. The summary statistics including the minimum, maximum, and mean concentration are described in Table 3. We found that the lowest mean concentration values for IL-6 were obtained in the control group (3.6 pg/mL), and the highest values in the ICU group (17.4 pg/mL), with a maximum value of 168.5 pg/mL. On the other hand, the lowest mean zinc values were obtained in the groups of COVID-19 patients who did not require hospitalization and control (105.3 μg/dL and 138.8 µg/dL, respectively), and the highest values in the ICU group (392 µg/dL).

Based on regression analyses, the following correlation values were determined: there was no significant correlation between serum IL-6 and serum zinc levels in SARS-CoV-2–positive cases (−0.0511, *p* value = 0.6366). Furthermore, zinc levels were negatively correlated with IL-6 in both the COVID-19 patient groups that did not require hospitalization and the control group, but the correlation was not statistically significant (Table 4).

Regarding hematological and biochemical parameters, the results of patients with SARS-CoV-2 hospitalized in the ICU and those hospitalized who did not require ICU were compared. It was found that lymphocyte values were significantly lower in patients with SARS-CoV-2 who were admitted to the ICU and that CRP and lactate dehydrogenase (LDH) values were significantly higher in this group, (Table 5) who also had higher IL-6 and zinc values compared to the other groups.

## 4. Discussion

In some cases, the infection caused by SARS-CoV-2 triggers an exuberant inflammatory response known as a “cytokine storm” [21]. It has been established that high levels of IL-6 are associated with greater clinical severity in patients with COVID-19 [7, 22–24]. In this sense, a meta-analysis carried out in 2020 that evaluated 8719 COVID-19 patients with severe disease found that those patients hospitalized in the ICU had elevated levels of acute phase reagents, such as erythrocyte sedimentation rate, CRP, IL-6, and IL-10, among others [25]. These findings are consistent with our results, which indicate high IL-6 concentrations in ICU patients. This fact is the basis for the use of drugs that act on inflammation. For example, tocilizumab, a monoclonal antibody against the IL-6 receptor, has been used to treat severe COVID-19 [7, 10].

On the other hand, zinc stands out as a potential therapeutic agent to treat patients with COVID-19 due to its immune regulation properties [13, 26, 27]. This trace element has antiviral properties and has a role in the inhibition of RNA and DNA synthesis, viral proteases, and viral replication [27]. Similarly, it plays an important role in many processes of immune development and immunomodulation, including B-cell and T-cell differentiation [28–30].

This study reports that COVID-19 patients in the ICU had the highest mean levels of IL-6 and zinc. In addition, the rest of the groups under evaluation had lower levels of both biomarkers. Our findings differ from previous studies, in which COVID-19–positive patients and severe patients were mostly zinc deficient. For example, Jothimani et al. [28] reported that COVID-19 patients had lower zinc levels compared to healthy controls. Furthermore, 57.4% of COVID-19 patients were found to be zinc deficient and developed more complications, prolonged hospital stays, and higher mortality [28]. Likewise, another study found that zinc concentrations were lower in patients with COVID-19 compared to healthy controls. Moreover, patients with lower zinc levels had a higher chance of mortality [31]. This possible discrepancy may have a temporary cause in relation to the moment in which the sample is taken to determine concentrations, particularly zinc due to its complex relationship with the molecular mechanisms in which it is involved in the immune response. It is known that after activation of the immune system, immune cells undergo a change in zinc homeostasis in the cytoplasm through zinc transporters. This bioavailable cytoplasmic zinc participates in signaling pathways to help direct robust, appropriate, and self-regulated immune responses. For this, zinc has to cross the cell membrane from outside the cell [32, 33].

The relationship between IL-6 and zinc in the context of infections is complex. As previously stated, zinc modulates the immune system and decreases levels of proinflammatory cytokines [12]. However, an increase in IL-6 levels may also affect zinc transporters and alter their homeostasis [14, 28]. During the acute phase response to infection, plasma zinc concentrations usually decline. Zinc molecules are rapidly shifted into the intracellular compartments for protein synthesis and neutralization of free radicals [30]. However, zinc levels can also remain elevated because inflammatory cytokines, such as IL-6, can alter the expression of the ZIP14 receptor in hepatocytes and impair zinc homeostasis [34]. This three-way interaction between IL-6, zinc, and infection makes it difficult to assess the impact of these biomarkers in the context of COVID-19 severity. Similarly, it has been described that serum zinc levels are associated with levels of IL-6 and IFN-γ as demonstrated by our results but without any significant association with the duration of hospitalization or mortality [35].

The comparison of hematological and biochemical parameters between patients with SARS-CoV-2 admitted to the ICU and those hospitalized but not requiring ICU admission reveals significant differences that underscore the severity of the disease in critically ill patients [36]. The notably lower lymphocyte counts observed in ICU patients suggest a more pronounced lymphopenia, which has been associated with severe immune dysregulation and poorer clinical outcomes in COVID-19 [37]. In addition, the elevated levels of CRP and LDH in this group indicate heightened inflammatory responses and potential tissue damage, both of which are characteristic of severe COVID-19 [38, 39]. The significantly higher IL-6 levels further support this, as IL-6 is a key mediator of the cytokine storm often seen in severe cases. Interestingly, the increased zinc levels in ICU patients could reflect either a compensatory response to inflammation or the impact of therapeutic interventions, given zinc's role in immune function and viral inhibition [2, 21]. Zinc supplementation in severe cases has been reported to lead to a significantly lower risk of mortality compared to the control group [40]. In Peruvian healthcare facilities, clinical recommendations for enteral nutrition for patients with COVID-19 infection in intensive care units indicate that a formula or supplement containing fiber and any other supplemental nutritional modules (protein pack, probiotics, fiber, zinc, etc.) can be considered and should be administered once a day at the discretion of the nutritionist [41]. This may explain the high zinc levels found in our study. These findings highlight the need for careful monitoring and targeted management of inflammatory and immune parameters in COVID-19 patients to improve outcomes [42].

In conclusion, it is reported that COVID-19 patients hospitalized in the ICU had higher mean levels of IL-6 and zinc compared to the other virus-infected groups and the control group. In this group, the highest values of lymphocytes, CRP, and LDH were also found with statistical significance compared to the group of hospitalized patients who did not require ICU. More longitudinal studies are required to determine the dynamics of these biomarkers and their relationship with disease outcomes.

## 5. Limitations

The main limitation of our study is that it was not able to determine the temporal relationship between the studied variables given the design of our study. It is not clear whether zinc causes IL-6 elevation or the other way around. Also, these biomarkers could be influenced by external factors, such as nutritional status and lifestyle habits. Finally, larger sample sizes could be useful for detecting more precise differences and associations. Hematological and biochemical analyses were only performed in groups of patients who received hospital care in the context of the COVID-19 pandemic.

## Figures and Tables

**Table 1:** Demographic characteristics of patients according to hospitalization status.

Characteristics^a^	Hospitalized		Not hospitalized	Control group *N* = 12 (%)	*p* value^∗^
	SARS-CoV-2 positive ICU *N* = 26 (%)	SARS-CoV-2–positive Non-ICU *N* = 26 (%)	SARS-CoV-2–positive Outpatient *N* = 24 (%)		
Age, years	57.0 ± 13.6	57.3 ± 13.4	55.1 ± 12.8	51.9 ± 11.9	0.6482
Age group, years					
25–39	2 (7.7)	2 (7.7)	2 (8.3)	2 (8.3)	0.999
40–49	6 (23.1)	6 (23.1)	6 (25.0)	3 (12.5)	
50–59	6 (23.1)	6 (23.1)	6 (25.0)	3 (12.5)	
60–69	8 (30.8)	8 (30.8)	8 (33.3)	3 (12.5)	
≥ 70	4 (15.4)	4 (15.4)	2 (8.3)	1 (4.2)	
Gender					
Female	6 (23.1)	5 (19.2)	12 (50.0)	8 (66.7)	0.007
Male	20(76.9)^*∞*^	21(80.8)^*∞*^	12 (50.0)	4 (33.3)	

Abbreviation: ICU, intensive care unit.

^a^Values are expressed as mean ± standard deviation or as number (percentage).

^∗^Fisher's exact test or Pearson's chi-square test or one‐way analysis of variance (ANOVA) test probability.

^∞^Univariate analysis using the polychotomous logistical model; significant *p* values are highlighted in bold (*p* value < 0.05).

**Table 2:** Clinical symptoms and comorbidities of patients.

Characteristics	Hospitalized		Not hospitalized	Control (*n* = 12)	*p* value^∗^
	SARS^+^ and ICU (*n* = 26)	SARS^+^ and non-ICU (*n* = 26)	SARS^+^ outpatient (*n* = 24)		
Symptoms at onset					
Cough	18(69.2)^*∞*^	17(65.3)^*∞*^	5 (20.8)	2 (16.7)	< 0.01
Expectoration	2 (7.6)	3 (11.5)	0 (0.0)	0 (0)	0.258
Fever	20(76.9)^*∞*^	11 (42.3)	6 (25)	1 (8.3)	< 0.01
Dyspnea	9 (34.6)	21 (80.7)	0 (0)	0 (0.0)	< 0.01
Fatigue	12 (46.1)	13 (50.0)	2 (8.33)	0 (0.0)	< 0.01
Anosmia	0 (0.0)	2 (7.6)	0 (0.0)	0 (0.0)	0.181
Diarrhea	0 (0.0)	0 (0.0)	0 (0.0)	0 (0.0)	—
Nausea/Vomiting	0 (0.0)	2 (7.7)	0 (0.0)	0 (0.0)	0.181
Headaches	3 (11.5)	3 (11.5)	8 (33.33)	2 (16.7)	0.153
Odynophagia	5 (19.2)	3 (11.5)	1 (4.1)	0 (0.0)	0.197
Comorbidities					
Diabetes	7 (26.9)	6 (23.0)	1 (4.1)	0 (0.0)	0.044
Hypertension	6 (23.0)	6 (23.0)	0 (0.0)	0 (0.0)	0.022
Obesity	9 (34.6)	3 (11.5)	0 (0.0)	0 (0.0)	0.001
Asthma	0 (0)	2 (7.6)	0 (0.0)	0 (0.0)	0.181
Coronary disease	1 (3.8)	3 (11.5)	0 (0.0)	0 (0.0)	0.197
Neoplasia	0 (9.0)	1 (3.8)	0 (0.0)	0 (0.0)	0.491
Chronic kidney disease	1 (3.8)	0 (0.0)	0 (0.0)	0 (0.0)	0.491
Others	5 (19.2)	6 (23.0)	0 (0.0)	0 (0.0)	0.031

*Note:* SARS^+^, cases of SARS-CoV-2–positive by RT-PCR.

Abbreviation: ICU, intensive care unit.

^∗^Fisher's exact test or Pearson's chi-square test or one‐way analysis of variance (ANOVA) test probability.

^∞^Univariate and multivariate analyses using the polychotomous logistical model; significant *p* values are highlighted in bold (*p* value < 0.05).

**Table 3:** Descriptive statistics for IL-6 and zinc in the study population.

Parameter		Hospitalized		Not hospitalized	Control group (*n* = 12)	*p* value^∗^
		SARS^+^ and ICU (*n* = 26)	SARS^+^ and non-ICU (*n* = 26)	SARS^+^ outpatient (*n* = 24)		
IL-6 (pg/mL)	Mean ± SD	35.2 ± 41.9	29.1 ± 35.3	5.7 ± 4.3	3.7 ± 0.8	< 0.01
	Max	168.5	134.6	17.7	4.9	
	min	4.7	3.3	2	2.7	
	IQR	35.6	28.6	2.1	1.45	

Zinc (μg/dL)	Mean ± SD	392.5 ± 657.6	200.6 ± 106.2	105.3 ± 57.6	138.8 ± 79.0	< 0.01
	Max	3402.5	491.8	218.1	324.0	
	min	32.7	53.3	6.2	9.1	
	IQR	129.5	117.7	88.3	86.8	

*Note:* A value of *p* < 0.05 was considered as statistically significant. SARS^+^, cases of SARS-CoV-2–positive by RT-PCR.

Abbreviations: ICU, intensive care unit; IQR, interquartile range; SD, standard deviation.

^∗^Kruskal–Wallis test and Dunn's post hoc analyses.

**Table 4:** Correlation analysis of IL-6 (pg/mL) and zinc (μg/dL) in patient and control group.

Pearson correlation (*p* value, sig. 2‐tailed)		SARS^+^ and ICU (*n* = 26)		SARS^+^ and non-ICU (*n* = 26)		SARS^+^ outpatient (*n* = 24)		Control group (*n* = 12)	
		IL-6	Zinc	IL-6	Zinc	IL-6	Zinc	IL-6	Zinc
SARS^+^ and ICU (*n* = 26)	IL-6	1	−0.2103 (0.3026)						
	Zinc	−0.2103 (0.3026)	1						
SARS^+^ and non-ICU (*n* = 26)	IL-6			1	−0.2936 (0.1455)				
	Zinc			−0.2936 (0.1455)	1				
SARS^+^ outpatient (*n* = 24)	IL-6					1	−0.1285 (0.5497)		
	Zinc					−0.1285 (0.5497)	1		
Control group (*n* = 12)	IL-6							1	−0.0169 (0.9585)
	Zinc							−0.0169 (0.9585)	1

**Table 5:** Hematological and biochemical parameters of hospitalized patient groups.

Hematological and biochemical parameters	SARS-CoV-2–positive ICU Mean ± SD	SARS-CoV-2–positive Non-ICU Mean ± SD	*p* value^∗^
Hemoglobin (g/dL)	14.65 ± 1.43	14.60 ± 1.87	
Leukocytes (/mm^3^)	11.00 ± 3.90	11.39 ± 4.57	
Lymphocytes (/μL)	789.62 ± 523.50	888.30 ± 849.41	*p* < 0.05
Platelets (10^9^/L)	328.81 ± 118.20	271.78 ± 119.80	
Glutamic-pyruvic transaminase (U/L)	100.96 ± 95.04	81.67 ± 53.26	
Creatinine (mg/dL)	1.11 ± 1.75	0.70 ± 0.18	
C-reactive protein (mg/L)	157.99 ± 124.49	213.32 ± 130.01	*p* < 0.05
Lactate dehydrogenase (UI/L)	304.62 ± 105.06	404.52 ± 133.21	*p* < 0.05
Procalcitonin (ng/mL)	0.32 ± 0.53	0.40 ± 0.51	
D-dimer (mg/L)	0.81 ± 0.91	0.96 ± 0.54	
Ferritin (ng/mL)	1232.62 ± 801.06	1303.97 ± 686.56	
Prothrombin time (s)	11.11 ± 1.51	11.45 ± 1.38	

^∗^Fisher's exact test or Pearson's chi-square test or one‐way analysis of variance (ANOVA) test probability.

## Data Availability

The data that support the findings of this study are openly available in figshare at https://doi.org/10.6084/m9.figshare.22911887.

## Nomenclature

ICU: Intensive care unit
Non-ICU: No intensive care
IL-6: Interleukin-6
ELISA: Enzyme-linked immunosorbent assay
SARS-CoV-2: Severe acute respiratory syndrome coronavirus 2
